# High-throughput, Efficient, and Unbiased Capture of Small RNAs from Low-input Samples for Sequencing

**DOI:** 10.1038/s41598-018-38458-7

**Published:** 2019-02-19

**Authors:** Cassandra D. Belair, Tianyi Hu, Brandon Chu, Jacob W. Freimer, Matthew R. Cooperberg, Robert H. Blelloch

**Affiliations:** 1 The Eli and Edythe Broad Center of Regeneration Medicine and Stem Cell Research, University of California, San Francisco, CA 94143 USA; 2 Department of Urology, University of California, San Francisco, CA 94143 USA

## Abstract

MicroRNAs hold great promise as biomarkers of disease. However, there are few efficient and robust methods for measuring microRNAs from low input samples. Here, we develop a high-throughput sequencing protocol that efficiently captures small RNAs while minimizing inherent biases associated with library production. The protocol is based on early barcoding such that all downstream manipulations can be performed on a pool of many samples thereby reducing reagent usage and workload. We show that the optimization of adapter concentrations along with the addition of nucleotide modifications and random nucleotides increases the efficiency of small RNA capture. We further show, using unique molecular identifiers, that stochastic capture of low input RNA rather than PCR amplification influences the biased quantitation of intermediately and lowly expressed microRNAs. Our improved method allows the processing of tens to hundreds of samples simultaneously while retaining high efficiency quantitation of microRNAs in low input samples from tissues or bodily fluids.

## Introduction

MicroRNAs (miRNAs) play a critical role in regulating gene expression in mammals. They have been found not only in tissue biopsies, but also in bodily fluids. A number of studies have pointed to miRNAs as biomarkers with potential utility in diagnosis, prognosis and treatment^1–4^. Sequencing provides an unbiased approach to miRNA biomarker discovery unlike qPCR or array analysis, which depend on validated probes and/or primers^5^. Until recently, cost was a substantial hurdle to large-scale screens by sequencing. That barrier has come down with increases in sequencing efficiency and the ability to multiplex samples. Now, it has become reasonable to sequence thousands of patient samples. However, accuracy and reproducibility remain major issues. The reasons for these issues are not entirely clear, but likely lie at the level of sample collection, RNA isolation, and library production.

Major issues plaguing small RNA library production include adapter ligation bias, adapter dimer contamination, PCR amplification bias and barcode bias^6–9^. All small RNA library production protocols involve the addition of an adapter or linker to each end of the RNA fragment of interest. The 3′ adapter enables reverse transcription, and together with the 5′ adapter, DNA amplification of the library as a whole. The 5′ adapter also provides the touch down sequence for the sequencing primer. 5′ and 3′ adapter ligation can lead to sequencing bias for specific miRNAs. In particular, the RNA ligases used for adapter ligation exhibit both sequence and structural preferences^10,11^. The addition of a barcode in either adapter can worsen this problem^6,7,11^. A common solution to the barcode contribution to ligation bias is to introduce it at the PCR step^12^. However, adding the barcode at this later step requires processing all samples individually all the way through the PCR amplification step, making the processing of large sample sets costly and tedious. PCR bias is yet another concern given that some sequences can be more efficiently amplified than others. For example GC content can effect PCR amplification^13,14^. The addition of unique molecular identifiers (UMIs) can address this issue, but are rarely used in small RNA sequencing^6,15–17^.

Here, we aimed to optimize a sequencing library strategy that is high throughput yet minimizes ligation and PCR bias. We started with a method originally described by Tuschl and colleagues, which introduces a barcode into the 3′ adapter allowing pooling of samples after the very first step of library production^18^. This many-to-one sample handling strategy allows for the processing of dozens to even hundreds of samples simultaneously. However, the barcode and fixed adapter ends introduces a high risk of ligation bias and, therefore, we introduced optimizations to reduce this bias. Also, we optimized methods to reduce contamination of libraries with self-ligated adapters, significantly enhancing the number of usable reads following sequencing. Finally, by introducing unique molecular identifiers (UMIs), we show that preferential PCR amplification of specific miRNA does not appear to be a significant contributor to miRNA bias. Instead, stochastic capture of miRNAs at very low input concentrations requiring high PCR cycle numbers does lead to poor and irreproducible calling of miRNA expression levels, especially of intermediately and lowly expressed miRNAs. Together, our optimized high-throughput method of library production results in the highly efficient and reproducible capture of small RNAs for downstream sequencing.

## Results

### Optimization of small RNA library preparation

**Figure 1:**
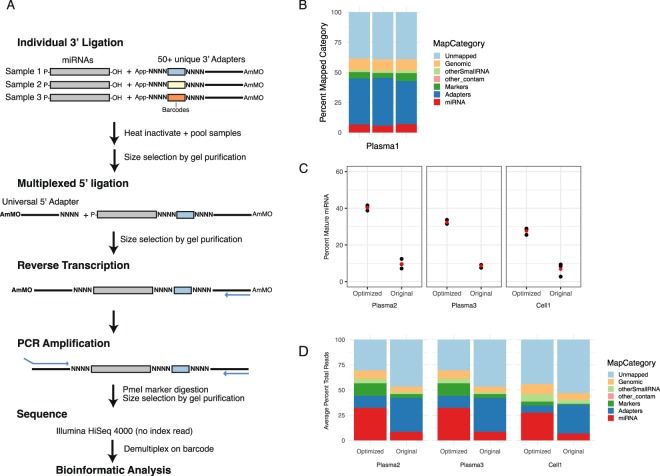
Modifications to high-throughput sequencing method improves capture of miRNAs. (**A**) Schematic of protocol to prepare miRNA libraries for sequencing. Modifications from original protocol noted in bold. (**B**) Percentage of different classes of RNAs captured from a plasma sample using the original conditions (0.85 μM 3′ adapter, 3.3 μM unmodified 5′ adapter). Ligations were performed in triplicate from the same RNA. Each replicate is shown as an individual bar. Note the low percentage of reads mapping to miRNAs (red). (**C**) Percentage of mature miRNAs captured using the optimized conditions (0.05 μΜ 3′ adapter, 0.33 μΜ amino-modified 5′ adapter) were compared to original conditions in three independent biological samples. The ligation reactions were performed in triplicate for each sample and protocol. Each replicate is shown as a black dot. Red dot represents average. (**D**) The average percentage of reads mapping to different classes of RNA for each sample and condition shown in C.

To improve upon the original Tuschl method^18^, we included two alterations that have been shown to improve ligation efficiency in other protocols (Fig. 1A). The addition of random nucleotides has been shown to increase ligation efficiency while also reducing ligation bias^5,19^. Therefore, we introduced four random nucleotides at the 3′ end of the 5′ adapter and the 5′ end of the 3′ adapter. In addition, we introduced 4 random nucleotides just 3′ to the barcodes to reduce risk of a fixed secondary structures. Together, these 12 random nucleotides also provide a unique barcode. The addition of polyethylene glycol at each ligation step has also been shown to improve ligation and thus was included in the protocol^5,20^.

In our experience, a major contributor to sequencing reads using traditional approaches of library preparation on low RNA input samples are the adapters themselves, especially 5′ adapter, consistent with ligation of multiple consecutive copies of the adapter (Fig. 1B). Therefore, we tested decreasing concentrations of 3′ and 5′ adapters starting with the concentrations used by Tuschl *et al*.; 0.85 μM and 3.3 μM respectively on RNA prepared from plasma. Titration of the 3′ adapter from 0.85 μM to 0.05 μM, holding the 5′ adapter steady at the original 3.3 μM, showed an increase from 7% to 18% miRNA reads (Supplementary Fig. S1). A titration of 5′ adapter from 3.3 μM down to 0.33 μM increased the percentage of reads mapping to miRNAs from 7% to 19% (Supplementary Fig. S1). There is evidence from Shore^20^ and others^21^ that blocking modifications on one or both adapters decreases adapter dimer formation. We next asked whether a blocking amino modification on the 5′ adapter could also increase the reads mapping to miRNAs, by suppressing ligation of 5′ adapter to itself. Blocking modification increased miRNA reads from 7% to 14% at the high concentrations of adapters (Supplementary Fig. S1). In all cases the increase in reads mapping to mature miRNA sequences came primarily at the expense of reads mapping to adapters (Supplementary Fig. S1). To confirm that the optimized conditions reproducibly result in a higher percentage of reads mapping to miRNAs, we prepared two library pools in parallel using either the original conditions or the optimized conditions using input RNA from two independent plasma samples (Plasma2, Plasma3) and RNA prepared from an established cell line, PC3 (Cell1). The combination of optimized adapter concentrations and 5′ modification led to a four-fold or better increase in the fraction of reads mapping to miRNAs in all samples (Fig. 1C). Analysis of the reads arising from these optimized conditions showed that the increase in mature miRNA reads again came primarily from a decrease in adapter sequences (Fig. 1D).

**Figure 2:**
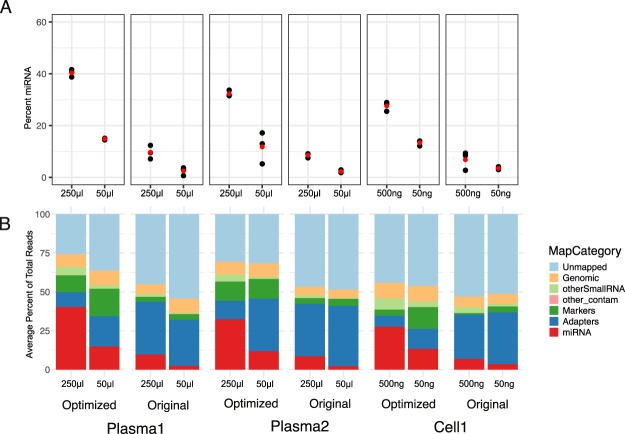
Improved miRNA capture also seen at low concentrations of input RNA. (**A**) The percentage of reads mapping to miRNAs at the indicated input following either the optimized (0.05 μΜ 3′ adapter, 0.33 μΜ amino-modified 5′adapter) or the original (0.85 μM 3′ adapter, 3.3 μM unmodified 5′ adapter) protocol. Black dots represent the three replicates from each input RNA for each sample, protocol, and concentration. Red dots represent average. (**B**) The average percentage of reads mapping to different RNA classes for each sample, protocol, and concentration.

Once the optimal adapter ligation conditions were determined, we explored the impact of input RNA concentration on miRNA capture efficiency. Based on previous titrations using independent samples, we repeated the highest and lowest inputs in the protocol comparison experiment. We found that capture was improved such that a similar percentage of miRNAs were captured using the optimized protocol at five to ten times less input than the original protocol (50 ul/250 ul Plasma 50 ng/500 ng Cell) in all three biological samples; Plasma 2, Plasma 3 and Cell1 (Fig. 2A), again due to a reduction in adapter contamination (Fig. 2B).

Examination of the miRNAs in the high input samples revealed that the optimized protocol identified over a hundred more miRNA species than the original protocol in each case (Plasma 2 976/844; Plasma 3 895/713; Cell1 1120/976). Thus, reduction of adapter contamination for low input RNA samples vastly improves quality of resulting small RNA libraries.

### Ligation Bias

**Figure 3:**
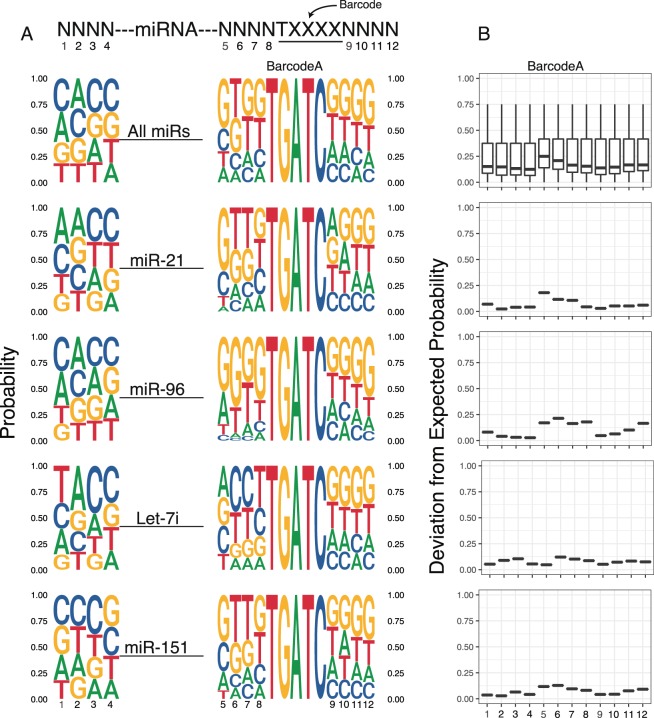
Nucleotide biases seen at ligation sites and varies between individual miRNAs. (**A**) SeqLogo representation of the base composition of the degenerate Ns over all miRNAs and select miRs representing high (miR-21) mid (Let-7i, miR-96) and low (miR-151) expression. The height of the letter representing the base is proportional to its probability. Bases 1–4 are at the 3′ end of 5′ adapter. Bases 5–8 are at the 5′ end of 3′ adapter. Bases 9–12 follow barcode in 3′ adapter. (**B**) Cumulative divergence from expected probability of nucleotide composition at each base across all miRNAs, and the same select miRs as (**A**). Data shown is from a 500 ng cellular RNA input sample.

Ligation bias can lead to differential miRNA capture^10,11,21^. The inclusion of random nucleotides at the ligated ends of the adapters allowed us to evaluate this bias, including which nucleotides and nucleotide positions within ligated adapters have the greatest influence on ligation bias for any particular miRNA. When averaged across all miRNAs, there was little evidence for a strong nucleotide bias at any position (Fig. 3A). However, examination of individual miRNAs showed substantial and distinct biases for particular nucleotides (Fig. 3A). For example, when annealed to BarcodeA, the highly expressed miR-21and the lowly expressed miR-151 showed a bias for GTT at positions 5 to 7, while the intermediately expressed miR-96, showed a bias toward G at position 5 and 6 (Fig. 3A). These biases were further influenced by the barcode (compare BarcodeA in Fig. 3A to BarcodeB in Supplementary Fig. S2). For example, with BarcodeB, miR-21 now showed a bias toward a C at position 5, miR-96 a bias for an A at position 5, and miR-151 a bias for C at position 5 (Supplementary Fig. S2). The intermediate expressed Let-7i also showed distinct adapter biases associated with each barcode (Fig. 3A, Supplementary Fig. S2).

Next, we asked how far nucleotide biases deviated from the expected equal distribution across all four nucleotides at each position. To do this we measured cumulative divergence of each nucleotide from its expected 25% contribution at each position. Looking across all miRNAs, the greatest bias was at positions 5 and 6 (Fig. 3B, top plot). However, once again this effect differed among individual miRNAs (Fig. 3B, lower plots). It also differed slightly between barcodes (Supplementary Fig. S2). Importantly, libraries produced using the same input, but different barcodes gave highly reproducible results (Supplementary Fig. S3, Supplementary Table 1). Together these data show that the usage of random nucleotides at the ligation ends of the adapter, especially the 3′ adapter, insures the unbiased and reproducible capture of all miRNAs even when using distinct barcodes to multiplex sample using the many to one sample handling strategy.

### PCR Duplication

**Figure 4:**
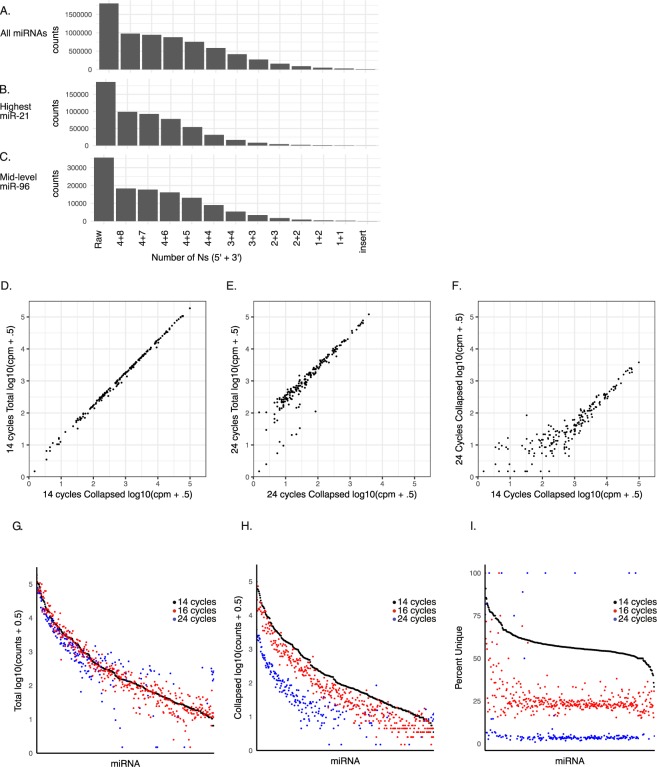
Unique molecular identifiers (UMIs) collapse duplicate reads and reveal linear relationship at low PCR cycles between total and collapsed counts, but drop out at high PCR cycles. (**A-C**) Effect of increasing length of UMI on number of miRNA counts following collapsing of miRNA + UMI, compared to total “raw” count. Number to left of + sign represents Ns on the 5′ adapter while numbers to right represent 3′ adapter. Insert represent collapse on miRNAs alone (i.e. without and UMI). Raw represents uncollapsed read count. Analysis shown for all miRNAs (A), a highly expressed miRNA (miR-21, B) and intermediately expressed miRNA (miR-96, **C**). (**D**) Correlation plot of log10 counts per million for each miRNA comparing collapsed versus uncollapsed (total) reads for a library amplified for 14 cycles. All 12 random nucleotides were used for collapsing. (**E**) Same as D, but amplified for 24 cycles showing reduction in correlation for low expressed miRNAs. (**F**) Same as D, but comparing only collapsed reads between library amplified for 14 versus 24 cycles, showing much poorer correlation for low to intermediate expressed miRNAs. (**G-I**) Direct comparison of read counts for each miRNA from libraries differing in the number of PCR amplification cycles. miRNAs are ordered from high to low expression in 14 cycle library. (**G**) Uncollapsed (total) counts per million. (**H**) Collapsed counts per million. (**I**) Percent unique reads (i.e. collapsed counts/total counts *100). Note noise created by high PCR cycle number on the lowly to intermediately expressed miRNAs. All libraries were made from one cellular input RNA.

Another potential source of bias in library production is differential PCR amplification of individual miRNAs. For example, it has been proposed that GC content can influence amplification^13,14^. Differential amplification could be further complicated by the specific pool of miRNAs existing in any one sample. Using the 12 random nucleotides in the adapter as unique molecular identifiers (UMIs), we asked whether differential PCR amplification exists and if so, how great is its impact. First, we evaluated whether the 12 random nucleotides provided a great enough diversity of sequences to cover the potential number of input miRNAs at our sequencing depth of over four million reads. If all positions are represented equally there are over 16 million combinations of the four bases at twelve positions, over 65 thousand at eight positions and 256 combinations at four positions. However, ligation bias reduces these numbers. Thus, to empirically determine how many random Ns are required to capture the full complement of input miRNAs, we evaluated the impact of increasing the number of random Ns on either side of the insert and collapsing resulting reads (Fig. 4A-C). The analysis was performed on all miRNAs combined as well as on two individual miRNAs, miR-21 and miR-96, representing a high and intermediate expressed miRNA, respectively. The miRNA counts showed a plateau starting at 10 Ns with miR-96 and 11 Ns for miR-21. Therefore, 12 Ns is predicted to capture the complete or near-complete complement of the most highly expressed miRNAs, even at a high sequencing depth.

Next, to determine whether there is preferential amplification of specific miRNAs, we compared total read counts to read counts collapsed using the full 12nt UMI. This analysis showed a linear relationship between total and collapsed reads indicating equal amplification across all miRNAs at low PCR amplification cycles (Fig. 4D). However, dilution (500×) of the same samples followed by high PCR amplification cycle numbers showed a decrease in the linear relationship (Fig. 4E). Comparison of collapsed reads from the 14 and 24 cycle runs showed an even greater difference (Fig. 4F). This later finding suggests stochastic capture at low input plays a more significant role than amplification bias. To further visualize the impact of input and PCR cycle number on expression, we plotted total counts, collapsed counts, and percent unique reads for each miRNA of a sample present in three libraries differentially amplified for 14, 16, or 24 cycles (Fig. 4G-I). Sixteen cycles overlapped well with fourteen cycles whether evaluating total or collapsed reads, even though the duplication rate was more than two-fold greater at 16 cycles (approximately 55% unique reads at 14 cycles versus approximately 25% unique reads 16 cycles). In contrast, 24 cycles showed overlap for the top third of expressed miRNAs, but than a dramatic drop off and large variation for the remaining miRNAs. The overlap in the more highly expressed miRNAs was quite surprising given the extremely high duplication rate (less than 5% unique reads). These findings show that the stochastic failure to capture miRNAs in low input libraries plays a much greater role than differential amplification in influencing the accuracy of evaluating miRNA expression values.

## Discussion

Here we describe an approach for analyzing the composition of miRNAs across many low-input samples in a sensitive, high-throughput and cost-effective fashion. A significant problem plaguing small RNA sequencing library production is that the adapter ligation can be inefficient, errant and/or biased resulting in sequencing data that does not accurately represent the ratios of miRNAs in the raw sample. These problems are amplified in low RNA input samples like plasma and urine^7^. Various strategies have been employed to increase efficiency of ligation including choice of ligase^10,11^, addition of PEG^5,20^, and addition of blocking modifications on either or both adapters^20^. Furthermore, a few studies have found that adapters designed with degenerate bases at the ends^18,19,21,22^ or internally^19^ reduce sequence bias in the ligation product. These strategies have resulted in step-wise improvements in sequencing. The method we present incorporates each of these improvements while using barcodes to allow sample pooling after the first ligation^18^. This many-to-one sample approach was further optimized to obtain miRNA expression data from human plasma samples.

There are few commercially available miRNA sequencing kits optimized for low-input RNA samples. The most commonly used kits (Illumina TruSeq, New England Biochemical’s NEBNext for example) recommend 1 ug of total RNA input. Trilink Cleantag^20^ and Bioo Scientific Nextflex^23^ have protocols for 1 ng input. With the optimized method presented here, miRNA libraries were successfully prepared from plasma samples with RNA levels undetectable by the Thermo Scientific Nanodrop One (<1.6 ng/μL) or Agilent Bioanalyzer RNA 6000 Pico kit ( < 50 pg/μL). The 5′ and the 3′ adapters included four degenerate bases at the ligation site of each. In addition to the blocking modification already present on the 3′ adapter^18^, a blocking amino modification was added to the 5′ end of the 5′ adapter. The concentration of each adapter was then titrated using the low input plasma samples to minimize adapter dimer contamination. These changes increased the efficiency of miRNA capture in the library five-fold.

Unbiased miRNA capture across samples was achieved regardless of nucleotide preferences by particular miRNAs. Consistent with our findings, Giraldez *et al*. recently showed that the addition of four degenerate bases to adapters of the TruSeq protocol greatly improved miRNA capture^22^. However, their library production strategy is based on the individual preparation of samples. Our use of the many to one sample approach greatly simplifies the building of libraries from large numbers of samples. In addition, it enables the combination of many low input samples.

Importantly, we show that increasing the number of PCR cycles to compensate for low sample input results in high duplication rates and loss of information for lowly and intermediately expressed species. Thus, combining multiple low input samples through early barcoding not only increases efficiency of library preparation, but also reduces artifacts associated with high PCR cycle number. A disadvantage of this pooled preparation is the potential for over-representation or under-representation of reads from a subset of samples. This can especially be the case when it is difficult to quantify and normalize RNA levels between samples prior to building of libraries. However, we have found it straightforward to rescue the under-represented samples, by repeating their sequencing in a separate pool with other lowly represented samples.

Our optimizations allow for robust screening of miRNAs from low-input biofluids of tens to hundreds of patients simultaneously. These methods should allow for increasingly accurate quantification of miRNAs in blood and tissue samples enabling the discovery of novel biomarkers of disease. Future improvements in RNA extraction and size selection methods will further enhance the utility of this method for small RNA biomarker discovery.

## Methods

### Ethics Statement

Blood samples were collected, and plasma prepared by standard clinical protocol between 2015 and 2016. Informed consent was obtained from all subjects. All samples were de-identified therefore this study was exempt from review by the UCSF Committee on Human Research.

### Sample RNA preparation

Cellular RNA was collected from PC3 cells that were obtained from ATCC (ATCC® CRL-1435) and cultured following recommended conditions. RNA was prepared by lysing the cells in Trizol followed by purification using the miRNeasy kit (Qiagen 217004) Biological replicates of PC3 extractions are referred to as Cell1, Cell2. Concentrations were determined by nanodrop (Thermo Fisher Scientific). Low-input RNA was collected from human plasma that was collected following standard clinical procedures using EDTA-coated tubes (Becton Dickinson). The RNA was prepared by thawing the plasma on ice, then following the micro miRNeasy kit (Qiagen 217084) with the change of adding 5x the volume of QIAzol lysis reagent to the plasma and using a final elution volume of 7 μl of RNase/DNase free water per 200 μl input plasma. Nanodrop One (Thermo Fisher Scientific), Bioanalyzer RNA 6000 Pico kit (Agilent) or Qubit (Life Technologies) analysis was employed to assess RNA quantity and quality. RNA from 200 μl of plasma was inconsistent or undetectable by any method. Biological Replicates of plasma samples from independent blood draws of healthy volunteers are referred to as Plasma1, Plasma2 and Plasma3.

### Small RNA-seq

Small RNA-seq libraries were made following the general workflow of Hafner *et al*. (Fig. 1A^18^). Input RNA for the cellular sample was 500 ng total RNA while input for the plasma samples was total RNA from 200 μl plasma unless otherwise noted. Sequencing adapters were synthesized by Integrated DNA Technologies using the codes provided in SupplementaryTable 2.xlsx. 3′ adapters were polyadenylated as described in the detailed protocol available in supplementary methods. The adapter concentrations were titrated as noted in the figure legends. 3′ adapters were used at 0.05, 0.2 or 0.85 μΜ while 5′adapters were added at 0.33, 1.0 or 3.3 μΜ. The experiments using the optimal conditions were done using 0.05 μΜ 3′ adapter, 0.33 μΜ amino-modified 5′ adapter. Fragments of the correct size were PAGE purified after each ligation step and after the PCR amplification. Unless otherwise noted, PCR was performed for 14 cycles. The 14-cycle and 24-cycle libraries presented in Fig. 3 were prepared from the same reverse transcription (RT) reaction. The cDNA was diluted 500 times with water prior to 24-cycles of PCR. Sequencing was performed on Illumina HiSeq 2000 sequencers. A detailed protocol is available at blellochlab.ucsf.edu/Protocols and in SupplementaryInformation.pdf.

### Small RNA-seq data analysis

The miRNA-Seq analysis data were preprocessed using CutAdapt v1.8^24^ to demultiplex and trim adapters, sequences were then aligned using STAR (params = ‘–genomeLoad NoSharedMemory–outReadsUnmapped Fastx–outSAMtype BAM SortedByCoordinate–outSAMunmapped None–outFilterMismatchNmax 0.05–outFilterMatchNmin 16–outFilterScoreMinOverLread 0–outFilterMatchNminOverLread 0–outFilterMultimapScoreRange 0–outFilterMultimapNmax 1–limitBAMsortRAM 1044427000’) or Bowtie v1.1.2 (-n 0 -l 18 –best) to a mature miRNA and other small RNA genomes were downloaded from miRbase^25,26^, and the human genome index curated by Illumina for bowtie or the comprehensive GENCODE annotations (Release 27 (GRCh38.p10) at https://www.gencodegenes.org/releases/current.html) for STAR. Reads were mapped sequentially to first remove all sequences inherent to library production (i.e. adapters, markers, PhiX), then mature miRNA, other small RNA, finally to the human genome. The remaining reads were labeled “Unmapped”. Analyses were performed and plots generated using custom R scripts in R studio^27,28^ and the tidyverse, forcats, ggseqlogo packages that are available for download on the CRAN website, https://cran.r-project.org. miRNA count data available in SupplementaryTable 1.xlsx; all data has been submitted to the GEO repository (GSE109162). Scripts are available upon request.

## Supplementary information

Supplementary Information

Dataset 1

Dataset 2

## Data Availability

GEO Accession: GSE109162. All raw sequencing data and processed counts data are available in GEO.
