# Supplementary material for: High-throughput, Efficient, and Unbiased Capture of Small RNAs from Low-input Samples for Sequencing
Source: Sci Rep. 2019 Feb 19;9:2262. doi: 10.1038/s41598-018-38458-7 (PMC6381177; doi:10.1038/s41598-018-38458-7)
Supplement: Supplementary file 1 — Supplementary Information [file 41598_2018_38458_MOESM1_ESM.pdf]

High-throughput, Efficient, and Unbiased Capture of Small RNAs from Low-input Samples for Sequencing

Cassandra D. Belair[1,2], Tianyi Hu[1,2], Brandon Chu[1,2], Jacob W. Freimer[1,2], Matthew Cooperberg[2], Robert H. Blelloch[1,2]

[1]The Eli and Edythe Broad Center of Regeneration Medicine and Stem Cell Research, University of California, San Francisco, CA 94143, USA.

[2]Department of Urology, University of California, San Francisco, CA 94143, USA.

Correspondence: Robert.Blelloch@ucsf.edu (RB)

SUPPLEMENTARY INFORMATION CONTENTS:

Supplementary Figure S1
Supplementary Figure S2
Supplementary Figure S3
Supplementary Methods

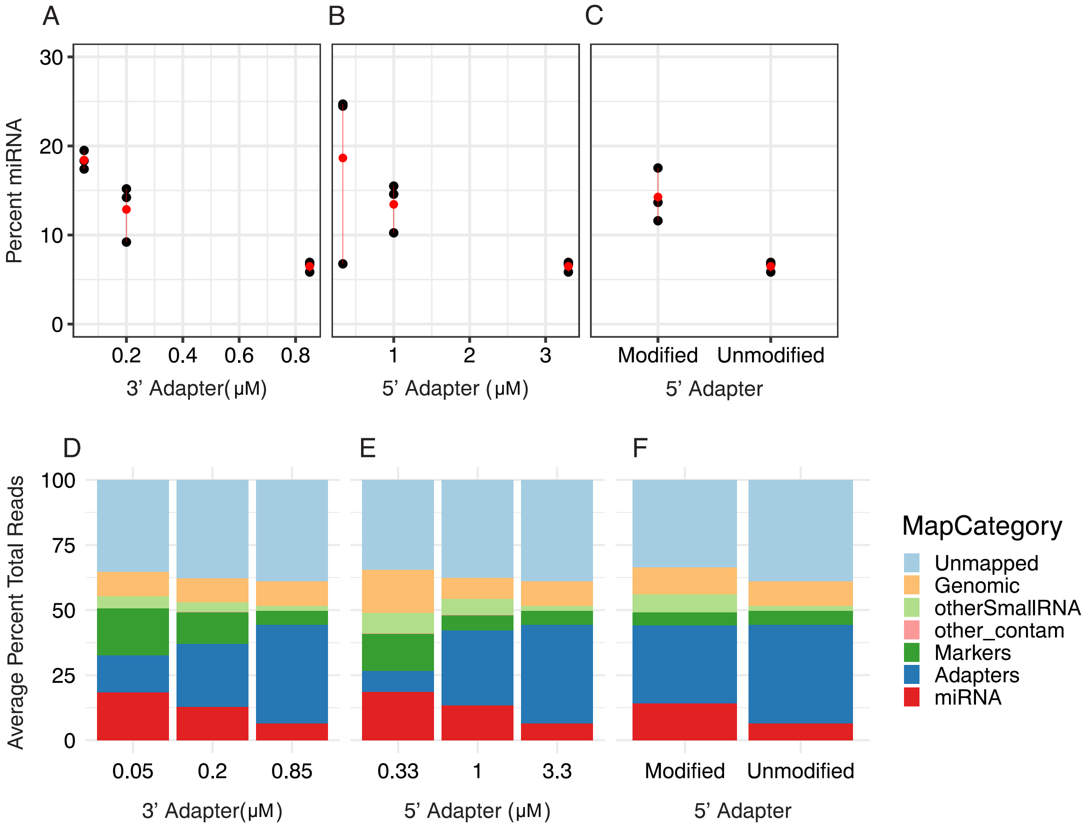

Supplementary Figure S1. Titrating adapters and adding blocking modifications improves miRNA capture and decreases adapter contamination. (A) Adapter concentrations were titrated to find the optimal concentration of each. Titration of 3′ adapter from 0.85 µM down to 0.05µM, holding 5′ adapter steady at 3.3µM. (B) Titration of 5′ adapter from 3.3 down to 0.33µM, holding 3′ adapter concentration steady at 0.85µM. (C) Addition of a blocking modification (5′ amino with C3linker) on the 5′ adapter also resulted in an increase in miRNA reads. Adapter was used at the high concentration for this experiment. (D-F) The average percentage of reads mapping to different classes of RNA for each condition in A-C as indicated. The ligation reactions were performed in triplicate from one plasma RNA (Plasma1).

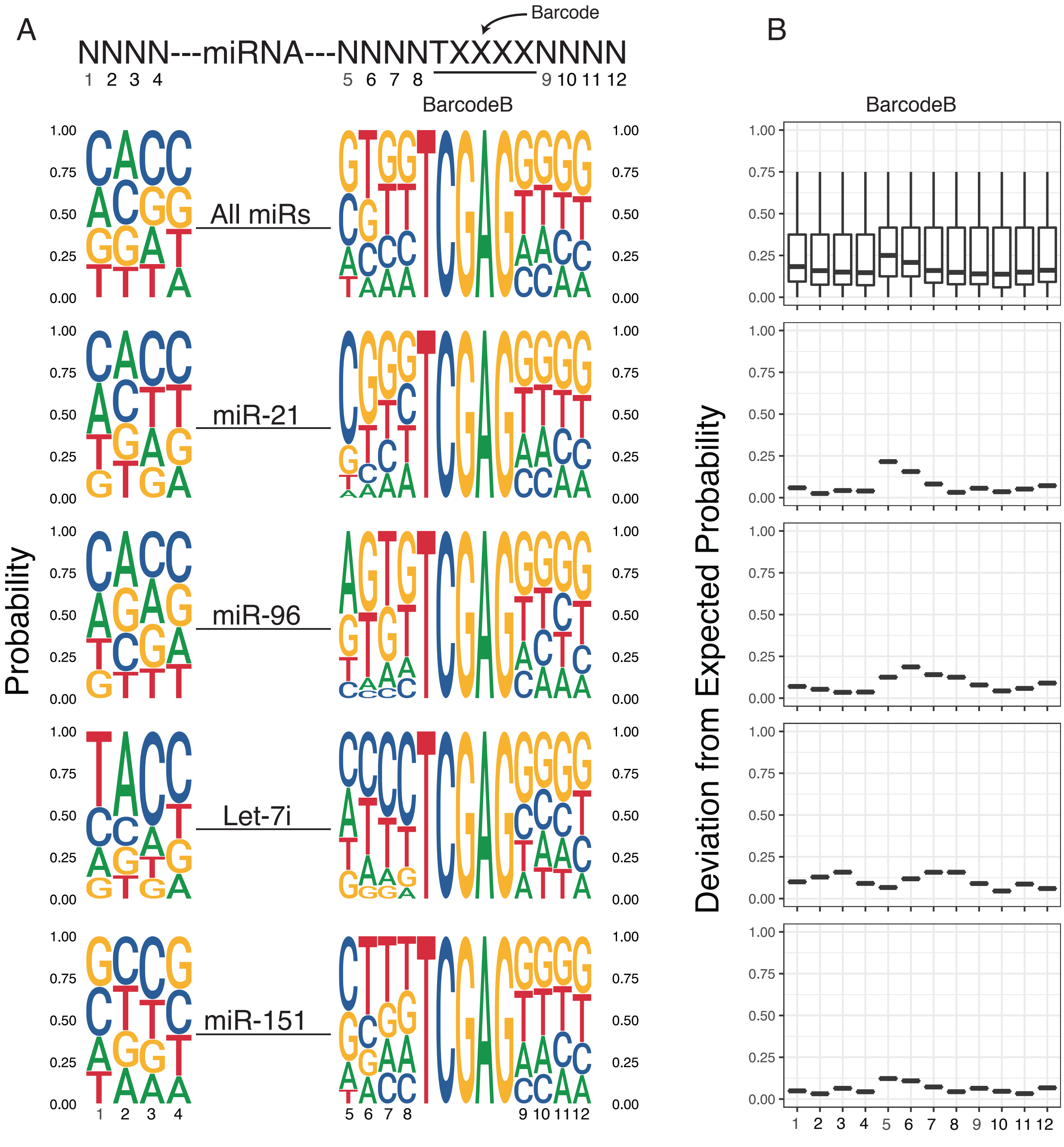

Supplementary Figure S2. Nucleotide biases seen at ligation sites and varies between individual miRNAs. (A) SeqLogo representation of the base composition of the degenerate Ns over all miRNAs and select miRs representing high (miR-21) mid (Let-7i, miR-96) and low (miR-151) expression. The height of the letter representing the base is proportional to its probability. Bases 1-4 are at the 3′ end of 5′ adapter. Bases 5-8 are at the 5′ end of 3′ adapter. Bases 9-12 follow barcode in 3′ adapter. (B) Cumulative divergence from expected probability of nucleotide composition at each base across all miRNAs, and the same select miRs as (A). Data shown is from same 500 ng cellular RNA prep as main figure 3, but using a distinct barcode (BarcodeB). Note effect of barcode by comparing this supplemental figure with figure 3.

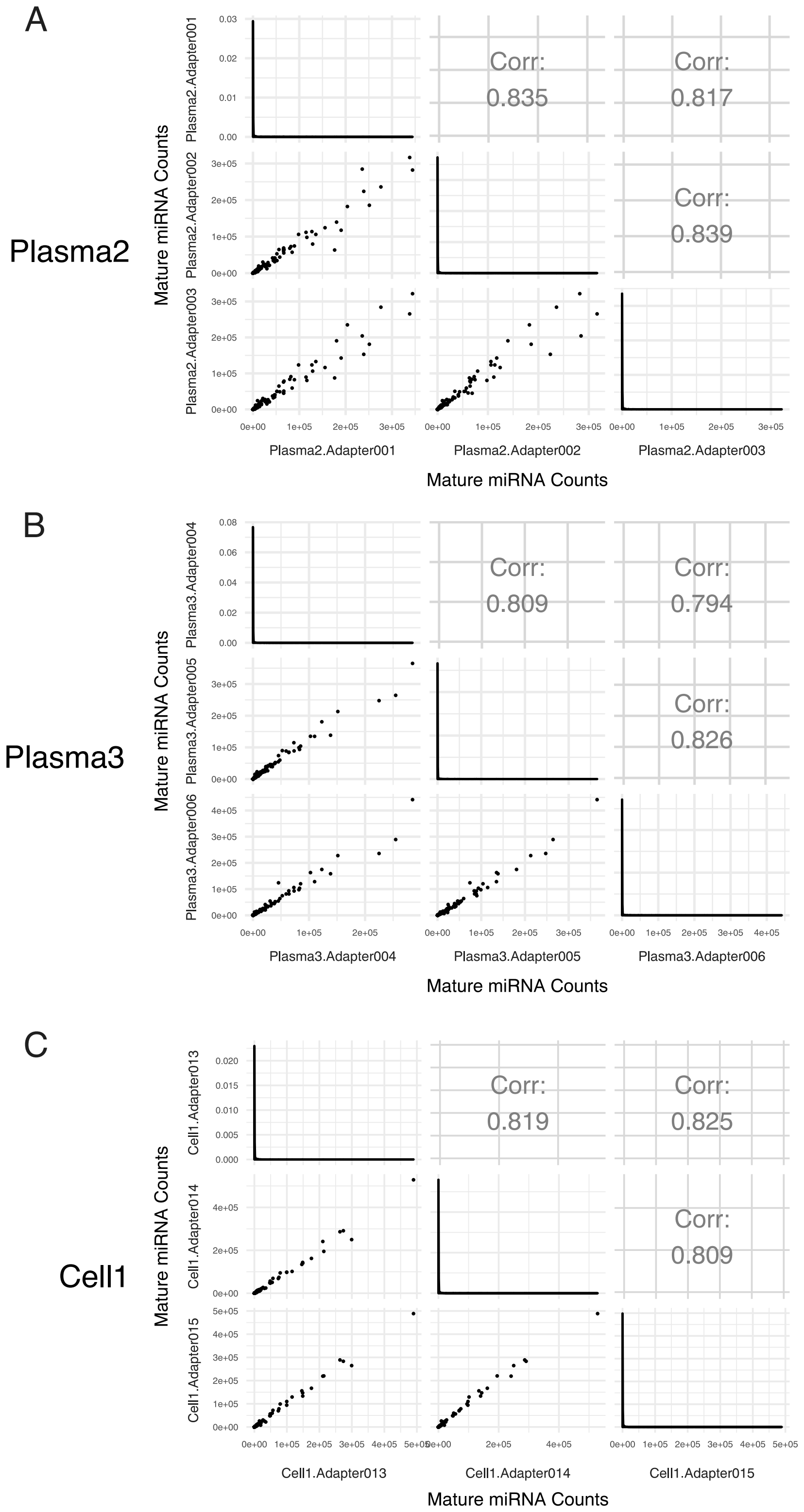

Supplementary Figure S3. Reproducibility of protocol using different barcodes, tested on three different samples. A) Correlation plots between three libraries using same plasma input RNA, but three distinct barcodes. B) Same as A, but with second plasma input RNA. C) Same as A, but with cell input RNA. Corr, Spearman Correlation. Uncollapsed raw read counts are shown.

Supplementary Methods:

High-throughput, Efficient, and Unbiased Capture of Small RNAs from Low-input Samples for Sequencing

Cassandra D. Belair[1,2], Tianyi Hu[1,2], Brandon Chu[1,2], Jacob W. Freimer[1,2], Matthew Cooperberg[2], Robert H. Blelloch[1,2]

[1]The Eli and Edythe Broad Center of Regeneration Medicine and Stem Cell Research, University of California, San Francisco, CA 94143, USA.

[2]Department of Urology, University of California, San Francisco, CA 94143, USA.

Correspondence: Robert.Blelloch@ucsf.edu (RB)

Table of Contents

Reagents: 3
Acrylamide Gel Reagents (used at each step): 3
3’ Adapter Adenylation: 3
Radio-label Marker Preparation: 3
3’ Adapter ligation: 3
5’ Adapter Ligation: 3
Reverse Transcription: 3
PCR: 3

Buffers: 4
10X TMD Buffer: (-20oC) 4
Denaturing PAA gel buffer: 4
10X TEN Buffer: 4
10X Orange G loading Buffer (100ml): 4
0.5M EDTA pH 8.0 (1L) 4
1M Tris pH 7.5 (1L) 4
10X TBE (5L): 4

Prepare Radio-labeled RNA Markers* 5

3’ Adapter Adenylation 6

Gel Extraction by diffusion: 7

Test Ligation for each new 3’ Adapter 8

Sample miRNA library preparation: 9

3’ Adapter Ligation 9

5’ Adapter Ligation 11

Reverse Transcription 12

PCR Amplification – Find Cycle Number 12
MinElute Reaction Cleanup Kit 13

Preparatory PCR 15
MinElute Reaction Cleanup Kit 15

Protocol initially based on:
Methods. 2008 January ; 44(1): 3–12. doi:10.1016/j.ymeth.2007.09.009.
Methods. 2012 October ; 58(2): 164–170. doi:10.1016/j.ymeth.2012.07.030.

Reagents:

Acrylamide Gel Reagents (used at each step):

10X TBE
Urea ultra pure (VWR RC-112)
40% Acrylamide (BioRad 19:1 #161-0144)
10% APS (freshly made, or frozen aliquots) (Bio-rad 161-0700)
2X PAA gel loading buffer
10X Orange G DNA Loading Dye

3' Adapter Adenylation:

200uM 4N-UMI 3' adapters with modifications /5Phos/ and /3AmMO/ (see xlsx sheet for barcodes)
/5Phos/ is 5' phosphate, /3AmMO/ is 3' amino modifier
50% PEG3000 in water (Sigma P4338, wt: 3350)
T4 RNA Ligase 1 (NEB M0204L) & 10X buffer
10mM ATP

Radio-label Marker Preparation:

200uM 19nt RNA marker (IDT)
rCrGrUrArCrGrCrGrGrGrUrUrUrArArArCrGrA
200uM 24nt RNA marker (IDT)
rCrGrUrArCrGrCrGrGrArArUrArGrUrUrUrArArArCrUrGrU
T4 Polynucleotide Kinase (NEB M0201S) & 10X PNK Buffer
EasyTides ATP gamma P32; 6000Ci/mmol, 10mCi/ml (Perkin Elmer BLU502Z250UC)
Zymo RNA Clean Up & Concentrator-5 (Zymo R1013)

3' Adapter ligation:

1uM Adenylated 4N-UMI 3' Adapter (unique adapter for each sample 2 sets of 50 available)
T4 RNA Ligase 2, truncated K227Q mutant (NEB M0351L)
10X TMD buffer
50% PEG3000 in water (Sigma P4338, wt: 3350)
RNaseOUT (Invitrogen 10777-019)
Radioactive 19nt/24nt RNA marker mix
3M NaCl
100% Ethanol
GlycoBlue (Ambion AM9516)

5' Adapter Ligation:

10X TMD buffer
10uM 5' Adapter (RNA) /5AmMC6/ is 5' amino modifier with C6 linker
(/5AmMC6/rGrUrU rCrArG rArGrU rUrCrU rArCrA rGrUrC rCrGrA rCrGrA rUrCrN rNrNrN)
10mM ATP
RNase Out (Invitrogen 10777-019)
50% PEG3000 in water (Sigma P4338, wt: 3350)
T4 RNA Ligase 1 (NEB M0204L)
Radioactive 19nt/24nt RNA marker mix
100uM 3' PCR primer (IDT)
CAAGCAGAAGACGGCATACGA

Reverse Transcription:

0.1M DTT
5X FSS buffer, (200mM Tris-HCl (pH 8.4), 500mM KCl)
10mM dNTP mix,
RNase Out (Invitrogen 10777-019)
SuperScriptIII Reverse Transcriptase (Invitrogen 18080044)
RNase H (*E. coli* 5U/ul, NEB M0297L)

PCR:

KAPA HiFi Hot Start 2X PCR Mix (KAPA KK2601)

10uM 5' primer (IDT) (AATGATACGGCGACCACCGAGATCTACACGTTCAGAGTTCTACAGTCCGA)
10uM 3' primer (IDT) (CAAGCAGAAGACGGCATACGA)
MinElute Reaction Cleanup Kit (Qiagen 28204)
Pme I Restriction enzyme (NEB R0560L) & 10X CutSmart Buffer
10bp DNA ladder (ThermoFisher/Invitrogen 10597012)
GlycoBlue (Ambion AM9516)
SYBR Gold (Invitrogen S-11494)

Buffers:

**All water is fresh from the MilliQ filtration system equipped with RNase exclusion filter

10X TMD Buffer: (-20oC)

0.5M Tris-HCl, pH7.4
0.1M $MgCl_2$
0.01M DTT

Denaturing PAA gel buffer:

98.8% formamide
1% v/v EDTA ph8.0
0.2% bromophenol blue

10X TEN Buffer:

0.1M Tris-HCl (pH 8.0)
0.01M EDTA (pH 8.0)
1M NaCl

10X Orange G loading Buffer (100ml):

40ml H_2O
30ml glycerol
10ml 1M Tris pH 7.5
20ml 0.5M EDTA pH 8.0
200mg Orange G

0.5M EDTA pH 8.0 (1L)

186.1g disodium EDTA·$2H_2O$
800 ml H_2O
~20g NaOH
adjust volume to 1000ml

1M Tris pH 7.5 (1L)

800 ml H_2O
121.1g Tris base
65 ml HCl (for Tris pH 8.0 45ml HCl)
when at RT adjust pH if necessary
bring volume to 1000ml with H_2O

10X TBE (5L):

540g Tris Base
275g Boric Acid
200ml 0.5M EDTA pH 8.0
H_2O to 5L

Prepare Radio-labeled RNA Markers*

*All procedures should be done according to specific Radioactive Use Authorizations of the University

1. Label 19nt and 24nt separately by making the following mix and incubating at 37°C for 30 min:

200uM Marker (19nt or 24nt)	5 ul	
10X PNK Buffer	1.5 ul	
Water	2 ul	
*Gamma P32-ATP	5 ul	
PNK	1.5 ul	
	**15ul total**	

2. Purify markers separately using Zymo Column Clean Up Concentrator (5). Elute each in 10ul and combine to yield 20ul marker mix.
3. Dilute working stocks 1:10 to 200ul with water. Aliquot and keep shielded in a designated freezer. Use within ~6 weeks.

3’ Adapter Adenylation

Note: We recommend purifying adapters by the gel diffusion method for best recovery, however the Zymo ZR small RNA PAGE recovery Kit (Zymo R1070) may also be used.

1. Make the following mix for each adapter and incubate at RT overnight:

	1 Adapter	X50
200uM Adapter	20 ul	
10X T4 RNA Ligase Buffer	10 ul	
10mM ATP	5 ul	
50% PEG3000	40 ul	
T4 RNA Ligase1	5 ul	
Water	20 ul	
	**100 total**	

Note: Store at -20 until ready to run gel.

2. Make a 7M Urea 20% PAGE gel to purify adenylated adapters (2 adapters/gel)
   a) Add together:

**Chemical**	**1 gel**	**2 gels**
Urea	21g	42g
10X TBE	5 ml	10 ml
Water	6.25 ml	12.50 ml
40% Acrylamide (bio-rad)	25 ml	50 ml

   b) Microwave 15s to dissolve urea. While mixture is cooling, prepare the gel sandwich. When cooled to RT, add the following an pour immediately:

10% APS	250 ul	500 ul
TEMED	37.5 ul	75 ul

3. Prepare un-adenylated control to run in each gel.

Unadenlyated Adapter	3 ul	
10X T4 RNA Ligase Buffer	2 ul	
50% PEG3000	8 ul	
Water	7 ul	
	**20ul total**	

4. Prepare adapters by mixing with 10X Orange G DNA loading buffer (12 ul or 3 ul). Boil at 90°C for 1 min and put on ice.
5. Flush wells and load adapters immediately. Split each adapter into 4 wells (~27ul each) leaving at least one empty lane between each adapter.
6. Run gel at 450V for 6 hours. Place gel on Saran wrap on a clean bench. Visualize using handheld UV light to see shadow. Cut out upper shifted adenylated adapter bands. Transfer gel bits to 0.5ml tubes previously prepared by poking 3-6 holes with 23G or larger needle from inside out.
7. Follow Gel Extraction by diffusion protocol on page 6.
8. Resuspend each purified adapter in 15ul ultrapure water.
9. Prepare 1uM aliquots for working stock.

Gel Extraction by diffusion:

1. Prepare 0.5ml flip-cap tubes for gel pulverization:
    a. Poke 3-6 holes in the bottom of a 0.5 ml tube by pushing a 23G needle from inside out.
2. put cut bands in the 0.5ml tube using a 2.0 ml screw cap tube for a collection vessel
3. add 400ul TEN buffer to the gel bits
4. spin the gel at 21,000 x *g* for 2 min. to pulverize gel
5. transfer any remaining gel bits to the screw cap tube by pouring and close cap securely
6. Incubate with constant mixing @ 37°C 1hr then reduce to 4°C o/n still mixing
7. Spin gel and buffer through Spin-X (0.22um Cellulose Acetate) column 15,000 x g for 2 min into a clean lo-bind tube to remove gel from TEN. Toss column with gel and keep flow-through
8. Add 1-2ul glycoblue to each tube.
9. Add 1.2ml (3x) vol 100% Ethanol (EtOH, room temperature), mix by vortex or inversion.
10. Freeze on dry ice for at least 30min (or store at -80°C)
11. Spin at max speed 4°C 30 min to precipitate
12. Discard supernatant, wash pellet with 1ml 70% EtOH, spin again max speed 10-20min 4°C
13. Carefully discard supernatant, quick spin to make sure all EtOH is removed.
14. air-dry pellet (~5-10min), don't over dry.
15. Re-suspend in ultra-pure water

Alternative Protocol ZR small-RNA PAGE Recovery Kit (Cat. R1070)
(not recommended because of lower yield)

Follow general guidelines to ensure the RNA isolation procedure is performed in an RNase-free environment.

1. Excise an RNA fragment from a PAGE gel and transfer the slice into a **Zymo- Spin™ IV Column** in a **Collection Tube**.
2. Crush the gel slice with a **Squisher™-Single** against the side of the column. Add 400 µl **RNA Recovery Buffer** directly into the column. Cap the column and incubate at 65oC for 15 minutes.
3. Quick freeze the samples on dry ice or in a -80ºC freezer for 5 minutes, then transfer columns back into 65oC for 5 minutes to thaw.
4. Snap off the Zymo-Spin™ IV Column tip and place the column back into a Collection Tube. Centrifuge at ≥1,500 x *g* for 30 seconds. Save the flow-through.
5. Transfer the flow-through from the Step 4 to a **Zymo-Spin™ IIIC Column** in a Collection Tube and centrifuge at ≥1,500 x *g* for 30 seconds. Save the flowthrough.
6. Add 2 volumes of **RNA MAX Buffer** to the flow-through from Step 5 and mix well.
7. Transfer the mixture to a **Zymo-Spin™ IC Column** in a Collection Tube. Centrifuge at ≥12,000 x *g* for 30 seconds. Discard the flow-through and place the Zymo-Spin™ IC Column back into the Collection Tube.
8. Add 400 µl **RNA Prep Buffer** to the column. Centrifuge at ≥12,000 x *g* for 1 minute. Discard the flow-through and place the Zymo-Spin™ IC Column back into the Collection Tube.
9. Add 800 µl **RNA Wash Buffer** to the column. Centrifuge at ≥12,000 x *g* for 30 seconds. Discard the flow-through and place the Zymo-Spin™ IC Column back into the Collection Tube.
10. Repeat Step 9 with 400 µl **RNA Wash Buffer**.
11. Centrifuge the Zymo-Spin™ IC Column at ≥12,000 x *g* for 2 minutes in an empty Collection Tube to ensure complete removal of the wash buffer.
12. Place the Zymo-Spin™ IC Column into a **siliconized DNase/RNase-Free Tube**. Add ~~6-15~~ **15 µl** of the provided DNase/RNase-Free Water directly to the column matrix and let stand at room temperature for 1 minute.
13. Centrifuge the Zymo-Spin™ IC Column at 10,000 x *g* for 1 minute to elute RNA. Recovered RNA can be used immediately or stored at ≤-70 oC.

Test Ligation for each new 3' Adapter

1. For each newly adenylated adapter prepare the following:

Water	9.9 ul	
10X TMD buffer	2 ul	
50% PEG3000	4 ul	
RNaseOUT	1 ul	
T4 Rnl2 trunc k227Q ligase	1 ul	
	**17.9 ul total**	
*Radioactive RNA marker mix	**1.5 ul**	
Test adapter (or water blank)	**1.0**	

   a. Make a master mix if testing multiple adapters. Aliquot 17.9 ul to each tube then add the Radioactive RNA marker and the adapter to be tested. One tube should have no adapter (marker alone).
   b. Incubate at RT 4h.
2. Heat-inactivate at 65°C for 20min and move to ice.
3. Make <u>7M Urea 15% PAGE</u> gel.

Urea	21 g	
10X TBE	5 ml	
Water	12.6 ml	

   Microwave for 15s to dissolve urea.
   While mixture is cooling, prepare the gel sandwich. When <u>cooled to RT</u>, add the following and pour immediately:

40% Acylamide	18.75 ml	
10% APS	250 ul	
TEMED	37.5 ul	

4. Add 20ul of PAA gel loading buffer to each sample, incubate at 90C for 1 min before putting on ice.
5. Flush the wells well and immediately load the gel with samples and unligated control (1ul marker, 19ul H_2O). Run samples at 350V for 4hours.
6. Wrap gel in Saran wrap and expose gel to a phospho-imager screen for 15-30min (depending on freshness of marker).
7. Confirm the shift of the radio-active marker in the adapter ligation lanes. If the marker does not shift, then start over with purification/adenylation of that adapter. If the marker does shift, then the adapter prep is considered good and ready for sample library prep.

Sample miRNA library preparation:

RNA preparation not included in this protocol:

- Total RNA purified by the Qiagen micro miRNeasy kit.
- Liquid samples are lysed in Trizol LS 1:3 or the included Qiazol at 1:5
- RNA should be prepared from equal plasma samples as the yield is typically below the limit of detection of the nano-drop, Qubit or Agilent pico RNA chip

3' Adapter Ligation

1. Prepare all samples with the final volume of 9ul (RNA+water). Include a marker-only control sample consisting of 1.5ul marker and 6.5ul water. Boil samples at 90C for 60s and then return on ice.
2. Add 1ul of a specific 1 uM 3' adapter to each sample, respectively (including marker-only control).
    a. Take care in noting which adapter will ligate to each sample.
    b. Also note that the 5' adapter ligation is standardized for a pool of 50 3'-adapted-samples. Scale the subsequent reactions down accordingly.
3. Then add the 10ul of following master mix to each of the tubes, using the repeat pipetor:

	1 adapter	x60	
Water	1.95 ul	117	
10X TMD buffer	2 ul	120	
50% PEG3000	4 ul	240	
T4 Rnl2 trunc k227Q ligase	1 ul	60	
RNaseOUT	1 ul	60	
*Radioactive RNA marker mix	0.05ul	3.0	
	**10ul total**		

4. incubate at RT for 4 hours
5. Heat-inactivate at 65°C for 20min and move to ice.
6. The marker-only ctrl should be frozen and used in the 5' adapter ligation gel extraction as a control.
7. Add 2ul of 3M NaCl to sample.
8. Pool up to 14 samples into a 2.0 ml screw cap tube. (for a 50-sample library, 3 pools of 12 and 1 pool of 14)
9. Add 1ul of GlycoBlue to each pool. Mix well.
10. Add 3X volume of 100% EtOH (720ul or 840ul) to each tube and mix well.
11. To precipitate, incubate tubes for at least 1hr on dry ice. Spin tubes at RT at max speed for 30 min. Remove supernatant, spin again and remove residual ethanol.
12. Air-dry pooled pellets for 5min.
13. Finish pooling up to 50 samples by resuspending the four pooled pellets sequentially in 20ul of water.
14. Gel purify the product to reduce carryover of unligated adapters and RNA to the 5' ligation reaction.
15. Add 20ul of PAA gel loading buffer and incubate at 90°C for 1 min then immediately on ice.
    a. unligated marker control = 1ul radioactive marker, 19ul H_2O, 20ul PAA gel buffer
16. Make **<u>7M Urea 15% PAGE</u>** gel.

Urea	21 g	
10X TBE	5 ml	
Water	12.6 ml	

Microwave for 15s to dissolve urea, when the gel sandwich is ready add:

40% Acylamide	18.75 ml	
10% APS	250 ul	
TEMED	37.5 ul	

17. Flush the wells well and immediately load the gel with unligated marker control and 3'-adapted-samples, leaving at least 2 lanes as a spacer around the samples.
18. Run samples at 350V for 4hours. (1400 volt-hours total, not more than 350V)
19. Wrap gel in Saran wrap and use Sharpie dipped in radioactive-marker to draw major and minor hash lines 1cm apart on the edges of the gel to serve as reference points during gel excision. Cover radioactive markings with Saran wrap and expose gel to a Phosphor-imager screen for 30min.
20. Use hash marks to excise gel containing the shifted markers. These should correspond to bands at 46 and 51bp. Re-expose gel to ensure precise excision. Re-cut if necessary
21. Follow the Gel extraction by diffusion method on page 5. Resuspend the precipitate in 8ul RNAse-free ultra-pure water.

5’ Adapter Ligation

1. Boil the purified 3’ adapter-ligated library at 90°C for 1min and then put on ice.
2. Add the following mix to the 8 ul 3’ Adapter ligation product and incubate 37°C for 2h:

	(50 pool)	
10X TMD buffer	3 ul	
10uM 5’ Adapter (RNA)	1 ul	
10mM ATP	3 ul	
RNaseOUT	1 ul	
50% PEG3000	12 ul	
T4 RNA Ligase1 (not truncated)	2 ul	
	**22 ul total**	

Sample may be frozen at -20°C after incubation.

3. Gel purify the reaction product to reduce carryover of unwanted products and unligated adapters.
4. Make 7M Urea 15% PAGE gel:

Urea	21 g	
10X TBE	5 ml	
Water	12.6 ml	

Microwave for 15s to dissolve urea, when the gel sandwich is ready add:

40% Acylamide	18.75 ml	
10% APS	250 ul	
TEMED	37.5 ul	

5. Add 20ul of PAA gel loading dye to each sample and boil for 1min at 90C then put on ice.
6. Load samples onto gel and include marker-only no-ligation control as well as marker-only 3’ ligation control that was previously saved. Leave at least one blank lane on each side of the library.
7. Run gel at 350V for 4 hours. (1400 volt-hours total, no more than 350V)
8. Wrap gel in Saran wrap and use radioactive-Sharpie to mark the edges. Then use phosphorimager to image gel.
9. Excise gel pieces in the shifted marker bands (71 – 76nt bands). Re-image to ensure proper excision of gel.
10. Follow the Gel extraction by diffusion method on page 6 with one modification. Add 1ul of 100uM 3’PCR primer prior to precipitation. This will act as carrier and will serve as the RT primer in the next step.
11. Resuspend the precipitate in 7ul RNAse-free ultra-pure water.

Reverse Transcription

1. Boil 5-prime adapter product (7ul) at 90C for 1min and transfer to a strip tube.
2. Add the following mix to each sample:

0.1M DTT	1.5 ul	
5X FSS Buffer*	3 ul	
10mM dNTP mix	4 ul	
RNaseOUT (40U/ul)	1 ul	
	**9.5 total**	

*200mM Tris-HCl (pH 8.4), 500mM KCl

3. Incubate samples at 50°C for 3min.
4. Add 0.75ul of SSIII RT (200U/ul) to each reaction and incubate at 42°C for 30min.
5. Add 1ul RNaseH to each sample and incubate at 37°C for 10min.
6. Add 79ul water, resulting in 95ul total volume

PCR Amplification – Find Cycle Number

It is recommended that if analysis will be done across multiple libraries, they are all amplified at similar cycle number.

1. First, test PCRs are used to determine optimal cycle number (12, 14, 16). Use the following mix (45 ul aliquot of master mix):

Water	17 ul	
2X KAPA HiFi Mix	25 ul	
10uM 5' primer	1.5 ul	
10uM 3' primer	1.5 ul	
	**50ul total**	
RT library product	5 ul	

2. Run the following thermo cycler program on the sample, varying the number of cycles.

95C	3:00min	Hold
98C	20secs	Cycle
65C	15secs	
72C	25secs	
72C	1:00min	Hold
4C	Forever	Hold

3. Purify using Qiagen minElute Reaction cleanup kit and elute in 10ul/PCR reaction. See Manufacturer's protocol on the next page.
4. Elute DNA with **10 µl RNase/DNase free water**

MinElute Reaction Cleanup Kit

(modified from manufacturer to refer only to centrifugation and to have volumes relevant to this protocol.)

- *This protocol is for cleanup of up to 5 µg DNA (70 bp to 4 kb) from enzymatic reactions.*
- *The yellow color of Buffer ERC indicates a pH of ≤7.5. Adsorption of DNA to the membrane is efficient only at pH ≤7.5.*
- *Add ethanol (96–100%) to Buffer PE concentrate before use (see bottle label for volume).*
- *All centrifugation steps are carried out at 17,900 x g (13,000 rpm) in a conventional tabletop microcentrifuge at room temperature (15–25°C).*
- *Refer to EN-MinElute-Handbook.pdf from qiagen.com for more details.*

1. Add 300 µl Buffer ERC to each PCR reaction and mix. The volume will be exceed the PCR tube, so mix into the column.
2. Check that the color of the mixture is yellow (similar to Buffer ERC without the enzymatic reaction). If the color of the mixture is orange or violet, add 10 µl 3 M sodium acetate, pH 5.0, and mix. The color of the mixture will turn to yellow.
3. Place a MinElute column in a provided 2 ml collection tube.
4. Apply sample to the MinElute column and centrifuge for 1 min. Discard flow-through and place the MinElute column back into the same collection tube.
5. Add 750 µl Buffer PE to the MinElute column and centrifuge for 1 min. Discard flow-through and place the MinElute column back into the same collection tube.
6. Centrifuge the column in a 2 ml collection tube (provided) for 1 min. Residual ethanol from Buffer PE will not be completely removed unless the flow-through is discarded before this additional centrifugation.
7. Place each MinElute column into a clean **siliconized** 1.5 ml microcentrifuge tube.
8. To elute DNA, add **10 µl RNase/DNase free water** to the center of the MinElute membrane. (Ensure that the elution buffer is dispensed directly onto the membrane for complete elution of bound DNA.) Let the column stand for 1 min, and then centrifuge the column for 1 min.

5. Digest the markers from the PCR reactions with PmeI at 37°C 1hr (or 4°C overnight):

cDNA	10 ul	
10X CutSmart Buffer	2.5 ul	
Water	12.25 ul	
PmeI restriction enzyme	1.0 ul	
	**25ul total**	

6. Analyze PCR reactions by running at <u>10% native PAGE</u> gel:

40% Acrylamide	12.5 ml
10X TBE	5 ml
Water	32 ml
10% APS	800 ul
TEMED	20 ul
	**50ml total**

7. Add 10X Orange G DNA loading dye to PCR samples and load the gel including the 10bp DNA ladder. DNA ladder must be diluted in correct buffers, following the chart below. This may be stored at -20°C.

RTR 10bp Ladder	2 ul	
10X CutSmart Buffer	2 ul	
Water	14 ul	
10X Orange G	2 ul	
	**20ul total**	

8. Run gel at 300V for 4 hours, (1200 volt-hours)

9. Stain with 1:10,000 SYBR Gold (diluted in TBE) to visualize bands.
    a. Make 50 ml of diluted SYBR Gold.
    b. Trim wells off gel to fit in white-lidded staining container, don't trim off the bottom.
    c. Place the gel in container on plastic wrap then add the pre-mixed stain. Cover and mix gently on the rotator protected from light ~15 min.
10. Visualize the Sybr Gold stained gel using the Bio-rad gel imager using the appropriate filter or blue LED light box.
11. Determine which PCR cycle number gives products between 116-121 while minimizing larger non-specific bands.
12. Look for the PME digested markers on the bottom of the gel.

Preparatory PCR

1. Prepare 3-6 50ul PCR reactions per library, using 5ul of cDNA template (45ul aliquot of master mix)
    a. Note: To increase the yield of cDNA needed for the sequencer increase the number of PCR reactions, not the number of PCR cycles.

	1 rxn	3.5 rxn
Water	17 ul	59.5
2X KAPA HiFi Mix	25 ul	87.5
10uM 5' primer	1.5 ul	5.25
10uM 3' primer	1.5 ul	5.25
RT library product	5 ul	
	**50ul total**	

2. Run the following thermo-cycler program using the cycle number previously determined.

95C	3:00min	Hold
98C	20secs	Cycle
65C	15secs	
72C	25secs	
72C	1:00min	Hold
4C	Forever	Hold

3. Purify PCR product using Qiagen MinElute column. Load 1.5-2 reactions per column. Elute with 10ul water per PCR reaction initially loaded.

MinElute Reaction Cleanup Kit

(modified from manufacturer to refer only to centrifugation and to have volumes relevant to this protocol.)

- *This protocol is for cleanup of up to 5 µg DNA (70 bp to 4 kb) from enzymatic reactions.*
- *The yellow color of Buffer ERC indicates a pH of ≤7.5. Adsorption of DNA to the membrane is efficient only at pH ≤7.5.*
- *Add ethanol (96–100%) to Buffer PE concentrate before use (see bottle label for volume).*
- *All centrifugation steps are carried out at 17,900 x g (13,000 rpm) in a conventional tabletop microcentrifuge at room temperature (15–25°C).*
- *Refer to EN-MinElute-Handbook.pdf from qiagen.com for more details.*

1. **Combine the 3 PCR reactions into 2 samples** (ie. Add 25 ul from one reaction into each of the others)
2. Add 300 µl Buffer ERC to the PCR reaction and mix. The volume will exceed the PCR tube, so mix into the MinElute column (stored at 4°C) in the provided collection tube.
3. Check that the color of the mixture is yellow (similar to Buffer ERC without the enzymatic reaction). If the color of the mixture is orange or violet, add 10 µl 3 M sodium acetate, pH 5.0, and mix. The color of the mixture will turn to yellow.
4. Centrifuge for 1 min. Discard flow-through and place the MinElute column back into the same collection tube.

5. Add 750 µl Buffer PE to the MinElute column and centrifuge for 1 min. Discard flow-through and place the MinElute column back into the same collection tube.
6. Centrifuge the column in a 2 ml collection tube (provided) for 1 min. Residual ethanol from Buffer PE will not be completely removed unless the flow-through is discarded before this additional centrifugation.
7. Place each MinElute column into a clean **siliconized** 1.5 ml microcentrifuge tube.
8. To elute DNA, add **15 µl RNase/DNase free water** to the center of the MinElute membrane. (Ensure that the elution buffer is dispensed directly onto the membrane for complete elution of bound DNA.) Let the column stand for 1 min, and then centrifuge the column for 1 min.

4. Pool all MinElute cleaned PCR reactions. (30-60 ul total)
5. PmeI Marker Digestion 1hr at 37C (or overnight at 4oC).

cDNA	**30 ul**	**60 ul**
10X CutSmart Buffer	7.5 ul	15 ul
Water	33.75 ul	67.5
Pme I restriction enzyme	3.75 ul	7.5
	**75 ul total**	150 ul

6. Prepare 10% Native PAGE gel:

40% Acrylamide	12.5 ml	
10X TBE	5 ml	
Water	32 ml	

when the gel sandwich is ready add:

10% APS	800 ul	
TEMED	20 ul	

7. Add 10X Orange G DNA loading dye to PCR samples (8-15ul)
8. Load 20ul of 10bp DNA ladder diluted in correct buffers, following the chart below. This may be stored at -20°C.

RTR 10bp Ladder	2 ul	
10X CutSmart Buffer	2 ul	
Water	14 ul	
10X Orange G	2 ul	
	20 ul Total	

9. Divide the Sample equally across 3 lanes (~55ul per lane)
10. Run gel at 300V for 4 hours, (1200 volt-hours)
11. Stain the gel with 1:10,000 Sybr-Gold (in 1X TBE) 15-20 min at RT
12. Visualize using the Blue LED light to prevent shearing and mutations.
13. Excise the 115-120 bp bands. Be sure to exclude adapter dimers at ~90 bp. Note whether there is an absence of non-specific amplification and evidence of marker digestion at the bottom
14. Follow the Gel extraction by diffusion method on page 6. Resuspend the precipitate in 15ul RNAse-free ultra-pure water.

Quantification of Library

1. Perform Qubit dsDNA HS assay to determine library concentration. Note: We have the Qubit 2.0 fluorometer. Refer to Qubit_dsDNA_HS_Assay_UG.pdf for details.
4. Calculate molarity of library.
    a. Divide ng/ml value from Qubit by 77.025 to yield nM concentration
5. Make dilutions appropriate for sequencing.
    a. The sequencing Core requires at least 20ul of 3-10nM library.
    b. If insufficient material is recovered another set of PCR reactions may be performed and pooled.
6. Use 1ul for Tape Station to verify high quality library size.
7. If multiple libraries will be pooled in one lane, quantitate using the same platform, Qubit or Tape Station as each has inherent error.
    a. Note: Digital PCR can be performed to assess quality prior to sequencing.
